# The Nightingale School and St. Thomas's Hospital—II

**Published:** 1917-11-17

**Authors:** 


### THE MATRONS' AND SISTERS' DEPARTMENT.

### THE NIGHTINGALE SCHOOL AND ST. THOMAS'S HOSPITAL—II.

Special and Ordinary Probationers.

There have always been "special probationers" in the Nightingale Home. They pay in all £30 "towards the cost of their maintenance during the first year," and they sign an agreement to continue in the service of the hospital for three years. Ordinary probationers pay no premium. They are paid quarterly at the rate of £10 in their first year, and they sign an agreement to remain in the service of the hospital for four years. Except for this additional year's service they are in no way differentiated from the special probationers.

The Preliminary Course of Instruction.

The preliminary course lasts nine weeks, and all probationers must pass through this before being transferred to their regular quarters in the Nightingale Home. A good kitchen and lecture-room are reserved for the preliminary students in a part of the Nurses' Home, and here five sets of candidates are prepared every year. All the housework in this part of the establishment is done by the pupils themselves, save the cleaning of floors. They keep their own rooms clean and neat, taking turns at such parts of the domestic work as are done for the others as well as for themselves. The time-table, which we subjoin, gives a good impression of the days spent in this admirably organised department. The Preliminary Training School's syllabus of lectures embraces the following subjects: (1) anatomy and physiology, dealt with in thirteen lectures; (2) theoretical nursing, dealt with in twelve lectures; (3) hygiene, dealt with in twelve lectures; (4) practical nursing, dealt with in twelve lectures; (5) chemistry of food, dealt with in six lectures; and (6) ambulance, dealt with in six lectures. There are frequent examinations and test papers, and the abilities of the pupils, mental, moral, and physical, are thoroughly tested. The success of such work as this depends entirely on the personality of the sister in charge. It is the spade-work done in this preliminary training school under the direction of the sister, who is supported by a specially qualified assistant, that prepares the nurse to pass on to the full training of the Nightingale School.

Preliminary Training School, St. Thomas's Hospital.
No. 1 Time-Table.

*Monday.*

6.30. Called.

7.40. Breakfast.

8.0. Prayers.

Housework.

9.15. Rooms.

10.0. Lunch.

10.30. Cookery

1.0. Dinner.

2.0. Physiology lecture.

3.0. Off duty.

5.0. Tea.

6.0. Class on lecture.

7.0. Notes and study.

8.45. Supper.

9.15. Prayers.

10.30. Lights out.

*Tuesday.*

9.15. Bandage-making.
10.15. Lunch.
10.45. Cookery practice.
2.0. Nursing lecture.
3-5. Off duty.

*Wednesday.*

9.30. Hygiene lecture.
10.30. Lunch.
11-1. Bandaging and practical nursing.
2.0. Chemistry of food lecture.
3-5. Off duty.

*Thursday.*

9.30. Physiology lecture.
10.30. Lunch.
11-1. Off duty.
2.0. Ambulance lecture.
3-5. Cookery practice.

*Friday.*

9.15. Splint padding.
10.15. Lunch.
11-12. Bandage practice.
12-12.45. Study.
2.0. Hygienic lecture.
3-5. Off duty.

*Saturday.*

9.30. Nursing lecture.
10.30. Lunch.
11-1. Practical nursing and bandaging.
2.0. Extra cleaning.
3-5. Off duty.

*Sunday.*

8.40. Breakfast.
9.10. Housework.
10.30. Chapel. Lecture.
8.45. Supper.

Hours not otherwise specified, same as Monday.

Probationers in the Nightingale Home.

Candidates who pass their examinations satisfactorily after their nine weeks' course now enter the Nightingale Home and are admitted to two months' trial in the hospital wards. They are then, if found suitable under these new conditions, accepted for training as probationer nurses. They reside in the Nightingale Home, where all have a separate bedroom, materials for uniform, and an allowance for washing. The Home has been entirely reconstructed so far as the sleeping and general accommodation is concerned. But the old refectory and sitting-rooms built at the beginning have been preserved, and these are hallowed by many associations with the foundress, and are adorned by her bust and portrait, with other mementoes of the past. Many of the rooms command fine views over the river, and the accommodation is in all respects worthy of the institution.

The working day for probationers extends from 7 a.m., when they enter the wards, to 8.30 p.m., when they return to the Home for the evening recreation and supper. Out of this period of 13½ hours from 4 to 4¼ hours are allotted to exercise and to meals, leaving a working day in the wards and lecture-room of 9 hours.

Each probationer goes through a course in general nursing, given by the sister tutor, during this first year, together with frequent class-instruction in elementary physiology and anatomy in continuation of the teaching given in the preliminary school. The aim is to render the instruction thorough rather than deep at this stage, where so many new impressions are being received. A great deal of what may be called "consolidation work" is got through in this year, with the aim of grounding probationers in practical work until they become absolutely proficient. Monthly reports on the probationers' practical efficiency in the wards are sent to the matron by the sisters under whom they work. There are no examinations during this year, but all note-books are returned weekly to the sister tutor that she may watch and guide the pupils' progress in technical and theoretical knowledge.

(*To be Continued.*)

☛ The first article appeared in "The Hospital" of November 10, 1917.

